# Pharmacists’ perception of their challenges at work, focusing on antimicrobial resistance: a qualitative study from Bangladesh

**DOI:** 10.1080/16549716.2020.1735126

**Published:** 2020-03-05

**Authors:** Elisabeth Darj, Md Shah Newaz, Muhammad Hamid Zaman

**Affiliations:** a Department of Public Health and Nursing, NTNU, Norwegian University of Science and Technology, Trondheim, Norway; b Department of Obstetrics and Gynecology, St Olav’s Hospital, Trondheim, Norway; c Department of Women’ and Children’s Health, Uppsala University, Uppsala, Sweden; d Department of Biomedical Engineering, Boston University, Boston, USA

**Keywords:** Antimicrobial resistance, Bangladesh, qualitative study, retail pharmacists

## Abstract

**Background**: The indiscriminate use of antimicrobial medicines has contributed to the development of microorganisms’ resistance to antibiotics. Antimicrobial resistance (AMR) is a major global health problem and is most acute in countries that have a weak health care and regulatory system and a high burden of infectious diseases, such as Bangladesh. Due to shortages of physicians available to diagnose and prescribe appropriate antibiotics, people with ailments in Bangladesh are forced to contact various types of health care services, including retail pharmacies, that lack supervision by qualified medical personnel. It is unknown how pharmacists perceive the AMR situation and the related challenges they face.

**Objective**: The aim of the study was to understand retail pharmacist’s perceptions regarding AMR.

**Method**: A qualitative study design was used, and in-depth interviews were held with retail pharmacists in Dhaka.

**Results**: The participants demonstrated a variety of concerns regarding AMR. They considered that self-medication, old prescriptions, lack of regulations and supervision, and readily available antibiotics were the main factors that contributed to the misuse of antimicrobial medicines and the development of AMR.

**Conclusions**: To control the further spread of AMR in Bangladesh, there is a need to work at several levels of the health system simultaneously. Initiatives could include establishment of the ‘Model Pharmacy’ programme nationwide, increasing and widening the focus on AMR in pharmacists’ education, ensuring the implementation of government guidelines on AMR, increasing public awareness of the consequences of antimicrobial use, and surveillance and monitoring of regulations and progress. A multipronged strategy is necessary not only for better AMR control but also for investment in a system that is well equipped to provide equitable care in the face of both existing and new health challenges.

## Background

Antimicrobial resistance (AMR) threatens the effective prevention and treatment of infectious diseases. However, antibiotics are easily accessed in many settings and have the potential to be misused or cause adverse effects [1]. Without effective antimicrobials, other life-saving treatments such as chemotherapy, major surgery, organ transplant, and diabetes management will become riskier, potentially driving up health care expenditures and intensifying the pressure on already strained health care systems [1]. The rapid rise in AMR has impacted national environmental, social, and economic targets, which in turn poses a threat to achieving several of the United Nations’ Sustainable Development Goals [2]. According to one estimate, AMR causes 700,000 deaths annually around the world and is expected to increase sharply to 10 million deaths per year by 2050 [3]. In addition to the use of antimicrobial treatments in medical practice, both farming and agriculture affect food production and enhance the development of AMR [4]. Thus, controlling AMR requires partnerships through international collaboration across human and animal health sectors and the integration of social, economic and behavioural factors [1,4]. At a global level, communities living in poor conditions are expected to be hit hardest and be pushed into poverty by AMR, but the effects of AMR go beyond impacting public health and global economy [4–6]. Resistance against commonly used antimicrobial medicines is remarkably high in countries with no restrictions on their use [2]. For example, a study conducted in India revealed a very high consumption of antibiotics in public and private health care, especially of broad spectrum and newer antibiotics [7]. A study based on sales data from 76 countries found that consumption of antimicrobial medicine had increased 65% in the period 2000–2015 [8]. Furthermore, the WHO indicates that in lower-middle income countries (LMICs) prescribers lack sufficient knowledge of how antimicrobial medicines should be used [1].

A recent review of the role of pharmacists and the use of antibiotics has shown that appropriately trained pharmacists can be part of the solution to overcome the global challenge of AMR by educating patients to enable them to ensure optimal use of antimicrobial medicines [9]. In developed nations, the role of pharmacists has expanded to provide multifaceted services, resulting in improved health outcomes and reduced costs [10]. Strengthening and enhancing pharmacists’ roles in LMICs may potentially affect the AMR situation in favour of a positive outcome. Poor hygiene, malnutrition and high-density residential areas in LMICs lead to the increased transmission of resistant strains of microorganisms [11,12], and globally, Southeast Asia has the highest overall risk of AMR among the world regions listed by the WHO [1]. Bangladesh is classified as a LMIC and is densely populated, with 163 million inhabitants [13]. Due to rapid urbanization and increasing slum areas, Dhaka has insufficient hygiene and sanitation services, which has contributed to the spread of infectious diseases. The existence of unqualified health care providers is deeply rooted in the local Bangladeshi culture and serves as a bridge between the community and formal health care services [14]. The Pharmacy Council of Bangladesh is the monitoring authority for pharmacy education. Pharmacists with ‘A’ grade certification have a bachelor’s degree in pharmacy, whereas pharmacists with ‘B’ and ‘C’ grade certification are respectively awarded a diploma after two years’ training and a certificate after three months of training [15]. However, there are also many unqualified, untrained persons in charge of pharmacies. Retail pharmacists are salesmen who meet customers but their views on the AMR situation are unknown in the literature. For this reason, the aim of the study on which this article is based was to understand retail pharmacist’s perceptions of AMR in Bangladesh.

## Methods

### Study design

A qualitative study design with in-depth interviews was used to explore retail pharmacists’ perceptions of AMR and to encourage them to explain their knowledge and role in the AMR situation in Bangladesh. A topic guide covering these aspects was followed during the interviews.

### Study setting

The study was conducted in Dhaka, the capital of Bangladesh, during the course of two months in 2018. Dhaka is estimated to have a population of 18 million and has numerous retail pharmacies to support its growing population [13].

### Study participants

Retail pharmacists were invited to participate in the study. No specific exclusion criteria were set regarding age, gender, work experience, formal training, and educational background. However, it is not culturally accepted for women to work as pharmacists and therefore all participants were men. The recruitment process followed the snowball method and aimed for purposeful inclusion covering various age, education and length of work experience. The participants signed an informed consent form that contained information about the study; no financial inducements were offered. Twenty-four individual interviews were held in various parts of Dhaka city, including the slums and major residential areas.

### Data collection

The interviews were conducted in Bengali, the language that all participants were comfortable with understanding and speaking and in which they could easily express themselves. The interviews were voice recorded after permission had been obtained from the participants. The transcribed material was translated into English and then three of those English translations were translated back into Bengali to check for accuracy.

### Data analysis

We performed manifest content analysis, following Graneheim and Lundman, in an attempt to understand the meaning of the transcriptions [16]. The analysis was performed jointly by two of the authors. The transcriptions were read several times to enable us to become familiarized with the context and to identify their inherent value. Mindjet’s MindManager version 2019 was used to visualize information in a flowcharts, to identify meaning units in the interviews [17]. The meaning units were condensed and coded, and similar codes were collected together and filed into subcategories, and subsequently merged into categories. The process was repeated until agreement on the categories was reached among the authors.

## Results

The study included 5 pharmacists with ‘A’ grade certification, 4 with ‘B’ grade certification, 6 with ‘C’ grade certification, and 9 who were untrained. All of the interviewees were men, as due to cultural norms it was impossible to find any women pharmacists to interview. The pharmacists were working in different retail pharmacies and they were in the age range 18–45 years. The participants’ educational background varied, and they had received 10–18 years of education. Their work experience as a retail pharmacist ranged from 1 year to 29 years. Our analysis yielded seven subcategories, which were merged into three main categories (Table 1). In this article, the participants’ quotes are coded according their qualification grade, their designated interview number, and their work experience. For example, ‘2A, 2 years’ experience’ refers to Interviewee 2, with an ‘A’ grade diploma and 2 years’ work experience.

**Table 1.** Bangladeshi pharmacists’ perceptions of antimicrobial resistance by category and subcategory

Category	Subcategories
Being a pharmacist	Source of professional knowledge Challenges faced
Awareness of AMR	Understanding of the AMR situation Antimicrobial medicine misuse Treatment managed by a pharmacist
Control and monitoring of the AMR situation	Increase awareness Model Pharmacy concept

### Being a pharmacist

#### Source of professional knowledge

The participant pharmacists received information concerning medical treatment from various sources, such as formal education, representatives of pharmaceutical companies, other pharmacists, own experiences, the Internet, and/or mobile phone applications:

Who else is going to provide us with this information? These people from the company visit us whenever a new product comes onto the market, and they provide the necessary information. (1C, 10 years’ experience)

Some pharmacists relied on older colleagues’ knowledge and some claimed they had learned about antimicrobial medicines and treatment through experience:

I had to take over our family business … I am slowly starting to learn which medicine is working for which kind of situation. (2C, 5 years’ experience)

If you work in this profession for a long time, you will know that automatically. (4Untrained, 18 years’ experience)

The participants claimed there was no need to revisit their educational institution after they had completed their studies. They were confident that they could find all necessary information from the Internet. Social websites and mobile phone apps are becoming popular for medicine-related information in Bangladesh and the interviewed pharmacists considered them convenient and reliable sources of relevant information:

I don’t need to contact the university. Currently, most of the information is based online (5A, 2 years’ experience)

‘ABC’ is like other mobile apps. It has almost all the medicine-related information. (3Untrained, 1 years’ experience)

#### Challenges faced

The pharmacists said they were sometimes seen by customers as merely shopkeepers, not as qualified experts with a diploma or certificate, and they felt disrespected when they were questioned about their expertise. When they conveyed professional advice about medication, patients occasionally did not want to receive the information and responded in a negative way:

When I try to give them advice, they tell me ‘I have consulted a high-profile doctor about my problem … You probably don’t know more than him.’ (5A, 2 years’ experience)

Actually, work is not problematic, but challenging. When I work … patients consider me a shopkeeper … sometimes it creates an awkward situation. (3A, 5 years’ experience)

[… basically, general people don’t have any idea what this AMR is or what will happen in the future if they misuse antibiotics. I tried to lecture some of the patients about this topic, but I can’t talk with every single patient who visits the shop. (2A, 8 years’ experience)

### Awareness of AMR and treatment with antimicrobial medicines

#### Understanding the AMR situation

A wide range of knowledge and concerns about AMR was revealed during the interviews. Pharmacists with a diploma or certificate were evidently more knowledgeable than untrained pharmacists, and the latter exhibited greater ignorance, misuse of antimicrobial medicines and poor understanding about AMR. Some pharmacists held the view that customers were misusing antimicrobial treatment, for example by taking medicines when they lacked proper indications for treatment or by not completing a prescribed course of treatment. The pharmacists were worried that some microorganisms had already developed resistance against conventional medicines, and thought that physicians were prescribing more effective antimicrobial medicines than in the past:

[When the] antibiotic is not working properly, doctors either prescribe higher doses or a more powerful antibiotic. (9Untrained, 2 years’ experience)

One pharmacist voiced the opinion that persons from rural areas were more susceptible to AMR than were persons from urban areas, due to the inferior quality of antimicrobial medicines sold in the former areas:

Even people outside Dhaka who complete the full course of medication will develop resistance, due to the low potency of the medication. (2A, 8 years’ experience)

Some pharmacists considered that AMR was not a matter of concern for them. They merely tried to follow the physician’s prescription when dispensing antimicrobial medicines to patients:

It is not my concern. The doctor’s surgery is not near this shop, so it is not my concern. I try to sell antibiotics as per doctors’ indications. (1C, 10 years’ experience)

We like to see the prescription, but if the patient is able to recall the name of medicine, then we understand that the patient might recognize that medicine … Besides, it is not our responsibility to stop the sale of the medicine. Also, antibiotics are not poison that will kill you. (2Untrained, 7 years’ experience)

#### Antimicrobial medicine misuse

Self-treatment is common among citizens of Bangladesh for several reasons. According to the interviewed participants, people sometimes thought they recognized their infections and therefore wanted to use the same kind of medication as they had used previously. Some people preferred to avoid contact with physicians due to concerns about the cost and time taken. Moreover, some people made their own decisions about how long they would continue to take their medicine and tended to stop taking it after the symptoms seemed to have disappeared, regardless of advice to complete the course of medication. According to the pharmacists, when a patient’s condition was not complicated, they were more likely to skip the full course of antimicrobial medicine and stop taking medicine as soon as they felt well:

If the patient’s condition is really critical … they will purchase a full course of that medication … but they have a tendency of taking parts of the antibiotic course instead of taking the full course at a time. (2C, 5 years’ experience)

The pharmacists claimed that literate people were showing health consciousness and purchased full courses of treatment. However, they were unlikely to buy the full course if the course had a long duration:

For a three-day course, they buy a full course, but for seven days’ treatment, they usually take a few antibiotics the first time. Later, they come back if their problem persists. (4C, 10 years’ experience)

According to the interviewees, people who lived in poverty might avoid paying for a full course of treatment initially and instead wait to see the effect of the first day’s treatment. Alternatively, they might try to negotiate the price of the treatment. Additionally, the interviewed pharmacists said that their customers often believed that antimicrobial medicines were suitable for various infections and helped them to recover faster from health problems. In some situations, the patients came to the pharmacists with old prescriptions or a prescription belonging to another person and asked for a repeat prescription:

In our country, you can take almost 80% of all existing medicines without a prescription. (1B, 9 years’ experience)

… patients use antibiotics frequently. It is because people want to recover fast from their infection problem. (8Untrained, 2 years’ experience)

Evidently, the pharmaceutical companies were more involved with the retail pharmacists than was ethical. Some pharmacists admitted that they tried to influence customers to buy newly available antimicrobial medicines on the market instead of the medicines prescribed by their physician:

Sometimes, when a company representative visits us … [they tell us] they have launched a new antibiotic, which is more effective than conventional [ones] … When patients visit us with their prescription … we, too, tell them there are new antibiotics in the market that might work better. (9Untrained, 2 years’ experience)

We are becoming more commercial. The doctors do not follow the patients. They follow the company, which provides them with a financial incentive. They [prescribe] medication from that company. (5Untrained, 4 years’ experience)

Regardless of a patient’s or customer’s condition, the pharmacists hesitated to provide multiple antimicrobial medicines, even when prescribed by a physician. They understood it would create a financial burden for a patient, who might fail to complete the course of treatment:

I never provide multiple antibiotics to patients. If one antibiotic is not working, we move to powerful antibiotics … but I never [dispense] the two antibiotics at the same time. (2B, 29 years’ experience)

Broad-spectrum antibiotics such as cephalosporins and macrolides are used frequently for common infections despite the enhanced risk they carry of leading to AMR:

Yes, sometimes they [patients] come to us looking for medicine. Nowadays, Zimax 500 [azithromycin] is a common medicine. Everyone knows about this medicine. So, they don’t need our help or their doctor’s help. (4C, 10 years’ experience)

Some pharmacists said that physicians commonly prescribed antimicrobial medicines based on their previous experience and the patient’s condition without waiting for the results of diagnostic tests:

Doctors have a logic that patients don’t want to wait for the cultural test result or that a patient’s situation might become worse if they wait for the result. For this, doctors tend to prescribe an antibiotic based on the empirical situation. (4A, 20 years’ experience)

#### Treatment managed by a pharmacist

When the interviewed pharmacists recommended treatment for customers’ health problems, they tried to replicate physicians’ ways of prescribing antimicrobial medicines. Some of the pharmacists had good relationships with physicians outside the profession and contacted with them informally if a patient developed a complication. Other pharmacists reported that they provided treatment to customers who were unable to contact their physician when they had health problems:

Most of the time, I call my own doctor who is really helpful. He works at a hospital … I don’t provide any treatment that I don’t understand. When I face complications, I contact him. (5C, 15 years’ experience)

Sometimes they compare medicines with their doctor’s prescription … They notice that the medicine I provide them is the same type of medicine as provided by the doctor … so they don’t want to waste money on doctor’s check-up [fees]. (5Untrained, 4 years’ experience)

Although the pharmacists provided various medical services to their customers, some knew that they were not allowed to propose certain treatment to them:

Actually, we pharmacists do not have authority to provide treatment, but sometimes do that, due to business purposes. (4C, 10 years’ experience)

[A pharmacist] … can’t prescribe any [medication], he can only review the prescription and counsel the patients. (2A, 8 years’ experience)

### Control and monitoring of the AMR situation

#### Increase awareness

As AMR is a problematic issue in Bangladesh, some pharmacists have tried to raise awareness among customers during their consultations with them. They expressed concerns about public awareness of AMR, and agreed that without increasing awareness, the AMR issue could not be averted. One interviewee suggested the following ways to build up awareness among the general public:

There is no alternative but to increase public awareness. If we can spread the awareness over the radio or TV … If we can run a programme in different communities … we can develop the awareness. (3A, 5 years’ experience)

Some graduate pharmacists tried to provide information about health problems and antimicrobial medicines to patients that was additional to that provided on the patients’ prescriptions, while other pharmacists did not want to worry the patients unduly when they lacked knowledge of the patient or the prescribed medicine:

I don’t give them too much information because if they hear more information, they might be scared or confused about their situation. (5Untrained, 4 years’ experience)

Additionally, they held the view that an important role was played by the educational system and insufficient training provided for certification for the retail pharmacy profession, which led to lack of awareness on the part of the pharmacists:

At present, training lasts for three months, but it is not enough to learn different aspects of medication and AMR. (2C, 5 years’ experience)

#### Model pharmacy concept

The pharmacists were aware of the national government’s intention to improve the public health situation by introducing standard practices in pharmaceutical services. In recognition of the problems of the low quality of some antimicrobial medicines, the existence of fake medicines and antibiotics, and illegal business practices in the sales of medicines, the Government of Bangladesh planned to implement a ‘Model Pharmacy’ programme. Under the programme, the service would be provided, managed and supervised by pharmacists with ‘A’ grade certification, who should be present in the dispensing facility, while pharmacists with ‘B’ and ‘C’ grade certification might be able to assist with dispensing medicines under the supervision of the manager. The participants anticipated that the introduction of the Model Pharmacy would improve the quality of the services and supervision in retail pharmacies:

For so long, our government has been planning to start the ideal pharmacy, but … it has not been able to start the initiative of the ‘Model Pharmacy’ programme. So, later, if it proves to be successful, the government plans to improve its programme gradually. (1A, 2 years’ experience)

Doctor’s sometimes follow the law for prescribing, sometimes they don’t. We in retail pharmacy also have specific rules. A ‘Model Pharmacy’ must be run by an A-grade pharmacist who has to complete at least a bachelor’s degree and who needs to complete [formal] training, and he must be registered. (2A, 8 years’ experience)

## Discussion

AMR is one of the most acute global health challenges of our time [3]. Pharmacists, who can be regarded as gatekeepers between physicians and patients, have a role to play in dealing with the challenge, yet few studies to date have systematically aimed to understand the challenges faced by pharmacists and their awareness of AMR.

We noted significant differences in the interviewed pharmacists’ knowledge of AMR. At the same time, patients’ and customers’ requests to decide and select their own treatment without any consultations were widespread and the practice was supported by social norms. The pharmacists admitted to dispensing medicines at patients’ requests. The unfortunate combination of high demand for antibiotics and poor levels of awareness of AMR contributed to repeated inappropriate use of antibiotics. As in other Southeast Asian countries, Bangladeshi pharmacies are often patients’ first point of contact with the health care system and a preferred channel for purchasing antimicrobial medicines [18]. The retail pharmacies are easy to access, stay open for long hours and sometimes offer medicines on credit. It is estimated that in Bangladesh there are more than twice as many salespersons without medical education as there are educated physicians and nurses [18]. This implies that the shortage of qualified health care providers and unequal access to health care and medical treatment urgently requires attention from the Government of Bangladesh in order to have an impact on the AMR situation. Furthermore, the pharmacists provide additional health care services, such as injections, wound dressing, and vaccinations, which are not approved by the country’s health policy [19]. This means that the governmental regulations on the dispensing and sale of medicines are neither practised by retail pharmacists nor supervised and therefore need to be improved. Due to the costs of seeking health care from physicians and the fact that the retail pharmacists provide treatment with a high out-of-pocket payment system, poor people tend to use a previous prescription or someone else’s prescription [18,20]. The pharmacists in our study occasionally shared basic instructions about the antimicrobial medicines with their customers, but at times they felt disrespected as the information was often disregarded, which in turn meant they were less motivated to offer counselling.

The situation in LMIC countries other than Bangladesh has similar troubling patterns with regard to AMR. For example, in Egypt patients purchase antibiotics without prescription, and patients’ self-medication is an important driver for the overuse and misuse of antibiotics, which is enhanced by pharmacists’ practices [21]. In the aforementioned study, polypharmacy with respect to antibiotics was found problematic. By contrast, the interviewed pharmacists in Bangladesh evidently made their own decisions and did not distribute more than one antibiotic a time, regardless of the prescription. A study conducted in Peru, an upper-middle income country, revealed good theoretical knowledge of AMR among pharmacists, but low awareness of the local AMR rates [22]. In common with the findings from Peru, our study revealed that pharmacists in Bangladesh were of the opinion that patient pressure was a contributing factor in the development of AMR [22]. Furthermore, similar to findings in Egypt, Peru, Qatar, India, Sri Lanka, and Australia, our study participants called for more education and guidelines on how to use antibiotics appropriately [21–25]. The pharmacists in the aforementioned studies recommended more education and national policies requiring a physician’s prescription prior to the dispensing of antibiotics.

Studies from more developed regions than Southeast Asia have shown that local pharmacy services are underused resources and that professionals in the public health care can contribute effectively and extensively to achieve health agendas [9,10]. However, our findings differ from those from the aforementioned contexts, as the pharmacists in Bangladesh claimed that they either had not received any formal training or they had not received enough training. Bangladesh currently does not have a compulsory minimum educational requirement for pharmacists, whereas in the neighbouring country, India, a pharmacist needs education equivalent to a two-year diploma and praxis in order to work in a retail pharmacy [7]. The Bangladeshi pharmacists suggested that one avenue to combat AMR could be through improvements in the education system and training requirements at universities, but we (the authors) would argue that tackling resistance to antimicrobial medicines requires more than changes in higher education curricula. Prevention strategies, along with public awareness, surveillance, and international coalition, have to be a central feature, as suggested by O’Neill in a report published in 2018, and in the WHO report on options for action against the threat of AMR [3,26].

Some positive examples from other countries may help to guide policies in Bangladesh with respect to AMR. For example, in Thailand the government has committed to tackle AMR and has developed a national strategic plan that includes action by the ministries of public health, agriculture, and education, as well as by various organizations and local units [27]. The example from Thailand could be an example of a way forward for Bangladesh, where antibiotics are inappropriately prescribed and used [28].

An observation about physicians’ behaviour in Bangladesh is worth further attention. The interviewed pharmacists were of the opinion that physicians either impulsively prescribed broad-spectrum antimicrobial medicines to treat infections or they did not follow existing guidelines for antibiotic use, either of which carried a higher risk of patients developing AMR. The pharmacists often sold the same type of medication as prescribed by the doctors, thus making it more difficult to minimize the consumption of antimicrobial medicines. However, even in the case of what the pharmacists deemed were appropriate prescriptions, patients often purchased only a single day’s dose of an antimicrobial medicine as opposed to the entire course, which thus led to misuse of the medicine that was unseen by the physicians.

In Bangladesh it is strictly prohibited to broadcasting information about medicines in the media and pharmacists learn about new medicines mainly from the pharmaceutical companies. The interviewed pharmacists pointed out that companies often shared commercial information about medicines and tried to influence pharmacists to provide certain medicines to their customers, which the latter admitted to doing. The influence of the pharmaceutical industry on drug prescriber’s choice of treatment has been debated and the ethics of the practice are complicated, as marketing and sponsorship designed to promote drugs can be disguised as education. More rigorous regulation of the relationship and transparency between the pharmaceutical industry and those who prescribe and dispense antimicrobial medicines is required [29,30].

In Bangladesh, while the pharmacists’ motives for joining the profession might have differed, they understood their importance and wanted to provide high-quality pharmaceutical services to their patients and customers. A commonly held view was that if they were properly equipped and had sufficient education and training they would be able to use their knowledge to improve the health care services. In India, for example, ‘pharmacy weeks’ are arranged, with workshops and seminars, with the aim of increasing knowledge and building awareness among patients about pharmacists’ contributions to the health care services [31]. Such programmes could be similarly used to improve pharmaceutical services in Bangladesh.

The participants in our study looked forward to seeing outcome of the public–private partnership programme ‘Model Pharmacy’. The Government of Bangladesh has acknowledged the lack of antimicrobial knowledge among the country’s pharmacists and the low quality of some antimicrobial medicines. The purpose of the planned ‘Model Pharmacy’ programme is to reduce illegal business dealings with fake antimicrobial medicines, to improve the safety and quality of pharmacists’ information provided to patients, and to ensure that only pharmacists with ‘A’ grade certification will be certified and responsible managers of retail pharmacies in the future. The programme also aims to improve professionalism, the quality of the medicines, hygiene, private places for consultation with pharmacists with ‘A’ grade certification, and a variety of stock at reasonable prices, with the ultimate goal of reducing AMR [32,33]. However, none of the interviewed pharmacists had had any experiences of the ‘Model Pharmacy’ programme at the time when our study was conducted.

### Strength and limitations

Our study is the first qualitative study to explore retail pharmacists’ perceptions of AMR and the challenges they face when providing services to patients and customers. Previous quantitative studies have been carried out to determine the extent of AMR in Bangladesh, but have not investigated the perceptions and knowledge of pharmacists who sell antimicrobial treatments and their views on AMR. All of our interviews were conducted in the local language to enable the participants to express their thoughts effortlessly. The interviews were conducted with different categories of retail pharmacists, who had different educational backgrounds and experience. To ensure trustworthiness, we have described the study design, methodology, and analytical process, and have supported our findings with relevant quotes from the interviewed pharmacists. Moreover, collectively, we have substantial experience in conducting studies in LMICs.

Some participants had difficulties in describing their views on the topics discussed, which might have been due to inexperience in participating in interviews or to their lack of knowledge. To address this potential limitation, we held the interviews in a context in which the participants felt comfortable, but it might have been better to have interviewed them in a calmer environment, as the interviews were occasionally interrupted by urgent tasks in the retail pharmacies where they worked. Another limitation of the study is that only male retail pharmacists were included in the study. The reason for this gender bias is that according to the cultural norm in Bangladesh, women are not involved in the retail pharmacy profession. More information about retail pharmacists’ knowledge and practices might have been gained if the interviews have involved other types of health care personnel and customers, but due to the study design only retail pharmacists were recruited.

## Conclusions

We found poor levels of awareness and repeated misuse of antibiotics, supported by social norms, but also a desire among retail pharmacists to improve the AMR situation in Bangladesh. The interviewed pharmacists evidently understood the development of AMR but were often unable to describe its impact and consequences. Although they were interested in their work, they felt that they were sometimes disrespected, which impacted their motivation. To utilize the unique ability and position of pharmacists to combat AMR and to control the further spread of AMR in Bangladesh, there is a need to work at several levels, such as the planned ‘Model Pharmacy’ programme, public awareness, improved pharmacists’ education, including education in AMR, and the provision of national guidelines for surveillance and monitoring regulations and progress. A multipronged strategy is necessary, not only for better control of AMR but also for investment in a system that is well equipped to provide equitable care for existing and new health challenges.

## Paper context

Overuse of antibiotics is a global health problem. Twenty-four retail pharmacists in Bangladesh interviewed about their views on antimicrobial resistance demonstrated a variety of concerns regarding AMR. Self-medication, old prescriptions, lack of regulations, and readily available antibiotics were the main factors that contributed to the development of AMR. A multipronged strategy is necessary for better AMR control as well as investment in a system to provide equitable care for both existing and new health challenges.
